# Prevalence and Trends of Hepatitis C, Hepatitis B, and Human Immunodeficiency Viruses Over Half a Decade Among Healthy Blood Donors Across Sindh, Pakistan

**DOI:** 10.7759/cureus.58374

**Published:** 2024-04-16

**Authors:** Dur-e-Naz Jamal, Samra Waheed, Sundas Gul, Maria Ali

**Affiliations:** 1 Public Health, Sindh Blood Transfusion Authority, Karachi, PAK; 2 Transfusion Medicine, Regional Blood Centre, Karachi, PAK; 3 Transfusion Medicine, National Institute of Cardiovascular Diseases, Karachi, PAK

**Keywords:** pakistan, sindh, blood bank, replacement donors, voluntary donors, healthy blood donors, hiv, hepatitis b & c

## Abstract

Introduction: Pakistan has a high prevalence of viral hepatitis and these transfusion-transmitted illnesses (TTIs) pose a major hazard to the health of patients who need blood transfusions, which has a negative impact on the affordability and accessibility of safe blood products in underfunded or less strengthened healthcare systems. While selecting a donor for blood donation, he/she must be healthy enough to donate 500 mL of whole blood, but some of them who were considered the healthiest community were caught to be reactive while getting screened for hepatitis B virus (HBV), hepatitis C virus (HCV), and human immunodeficiency virus (HIV), which reflects the true prevalence of these illnesses in this specific population.

Objective: To study the seroprevalence and trends of HBV, HCV, and HIV in healthy blood donors of Sindh, Pakistan.

Subjects and methods: Blood donated by healthy donors from Sindh, collected from January 2018 to December 2022, was tested by enzyme-linked immunosorbent assay (ELISA) or chemiluminescent immunoassay (CLIA) at 185 blood centers running under the umbrella of Sindh Blood Transfusion Authority Pakistan (SBTA).

Results: The results of serological screening tests for HBV, HCV, and HIV performed from January 2018 to December 2022 revealed a continuously increasing trend of all infections. The total number of blood donations in the blood banks across the province showed a progressive increase from 22,822 donors in 2018 to 937,039 donors in 2022, which is 14.21% of the total increase. Among 4,199,195 donors screened from the said period, 3,821,268 (91%) were replacement donors while only 3,77,927 (9%) were voluntary donors. Among them 3,870,598 (92.2%) were males and only 3,285,97 (7.8%) were females, whereas with regard to donors’ age, most of them i.e. 2,664,648 (63%), fall in the 29-39 years age group. Overall, from 2018 to 2022, out of a total of 4,199,195 individuals screened, 81,266 (1.94%) tested positive for HCV, 71,688 (1.7%) tested positive for HBV, and 6,711 (0.15%) tested positive for HIV. The total number of positive cases across all three infections was 159,665 (3.80%). The overall average seroprevalence of hepatitis B surface antigen (HBsAg), anti-HCV, and anti-HIV among blood donors of 185 blood banks, for five years, was 2.78%, 3.82%, 3.65%, 4.15%, and 4.04%, respectively.

Conclusion: The study highlights a concerning increase in the prevalence of HCV, HBV, and HIV among blood donors in Sindh, Pakistan, over the five-year period. It underscores the importance of continued surveillance, prevention, and intervention strategies to address these infections. The recommendations include the promotion of voluntary blood donors and screening of donated blood through a highly sensitive screening assay (nucleic acid testing). There should be centralized blood collection systems having better personnel and equipment, and non-remunerated voluntary blood donations must be strongly encouraged. All these, however, require strong political commitment and multisector engagement with comprehensive policy implementation.

## Introduction

Blood transfusion is a common therapeutic treatment used to treat a wide range of illnesses, trauma, procedures, and blood-related disorders like thalassemia, hemophilia, and other hemoglobinopathies. However, transfused blood can be dangerous owing to contamination with high-risk common infectious illnesses such as hepatitis B and C viruses (HBV and HCV), which are the most frequent [1]. With 2 billion units of blood donated globally each year [2], blood donors are the assets of any country. Poorly screened blood, on the other hand, may pose a significant risk of transmitting blood-borne illnesses to the recipients.

The major issue in safe blood provision is a high seroprevalence of transfusion-transmitted illnesses (TTIs) in donated blood [3]. The risk of acquiring blood-borne diseases is higher in developing countries due to the higher prevalence of TTIs among the donors and the lack of poor practices in blood screening [4]. TTIs are mostly caused by human immunodeficiency virus (HIV), HBV, HCV, *Treponema pallidum* (the bacterium that causes syphilis), and malaria [5].

It is crucial to assess the prevalence and distribution of HBV, HCV, and HIV; as called silent killers, they are mostly asymptomatic and are in the latent phase, therefore increasing the trouble in diagnosis [6,7].

The past several decades have witnessed great advances in techniques for detecting these TTIs; with the advent of nucleic acid amplification technique (NAT), western countries have decreased the risk of TTIs to a major extent as NAT testing is able to identify those carriers who are in window period of the disease and can transmit disease actively. Despite this dramatic progress worldwide, Pakistan is still far from achieving a “zero risk” blood supply due to limited budget resources and lack of education and communication [8].

The safety of the blood supply is compromised, as the country still depends heavily on replacement donors, and the escalating costs of medical care make the desired result still more difficult to obtain. The yearly transfusion rate in Pakistan is approximately 1.5 million [9]. The World Health Organization (WHO) reports that two billion individuals worldwide are infected with HBV, 200 million are infected with HCV, and 33.4 million are HIV positive. According to the Centers for Disease Control and Prevention (CDC), HBV is 10 to 100 times more infectious than HCV and HIV, respectively [10], and HBV as well as HCV infection is steadily rising in Pakistan. It is estimated that around nine million people currently have HBV and around 10 million individuals are afflicted with HCV. The prevalence of HBV, HCV, and HIV in Pakistani blood donors has been found to be 2.33%, 3.78%, and 0.06%, respectively, in 2007 [9]. Pakistan has around 240 million population, bearing a significant socioeconomic burden because of the escalated frequency of such infectious diseases. In addition, there is a significant prevalence of anemia, communicable infections, malnutrition, thalassemia, obstetrical problems, and traumatic injuries [11].

The donors in Pakistan are mainly categorized into three groups: exchange or replacement blood donors (those who want the blood of the required blood group from the blood bank and donate blood in exchange); voluntary blood donors (those who voluntarily come to the blood bank and donate blood); and voluntary blood donors in the camps conducted outside the blood bank [11]. Donation or exchange transfusions are generally done in hospitals, with hospital blood banks handling the full hemovigilance procedure from donor to receiver, including but not limited to blood collection, screening, storage, crossmatching, transportation, and transfusion. In addition, there are several private blood banks around the country that provide inadequate quality control, poor screening methods, storage, and transfusion practices. Apart from the corporate sector, several non-profit organizations offer transfusion services to certain demographic groups, such as thalassemia and other hemoglobinopathies patients. Due to a high prevalence of blood disorders such as thalassemia and hemophilia, as well as the demands for hemodialysis, pregnancy, surgical procedures, accidents, and emergencies, there is a significant need for blood. The WHO recommends that all given blood be tested for HIV, HBV, HCV, malaria, and syphilis [2].

Due to lack of knowledge, deficient screening tests, lack of access to health facilities, and poor surveillance systems, the precise number of TTIs in our population is still unknown. Updating prevalence statistics on TTIs among blood donors can give insight into trends in blood-borne infections in blood donor populations, and, as a result, establish the safety of blood donations collected. The purpose of this study was to look at the trends in the prevalence of HBV, HCV, and HIV infections among blood donors in the Sindh province of Pakistan during the last five years (2018-2022).

This article was presented as a poster presentation at ISBT Cape Town 2023 and the abstract was published in Vox Sanguinis on November 9, 2023 (DOI: 10.1111/vox.13534).

## Materials and methods

A retrospective cross-sectional study was conducted from January 2018 to December 2022 among 4,199,195 blood donors aged 18 to 60 years at the different blood banking centers which are supervised and regulated by the Sindh Blood Transfusion Authority (SBTA). The replacement donors were family members, friends, or relatives of the patients concerned. SBTA provides 185 blood banks to certain healthcare facilities of 28 districts with over 47 million inhabitants of Sindh and adjoining areas. Potential donors undergo a questionnaire and a physical examination performed by trained physicians, with donor eligibility criteria of being apparently healthy, being between 18 and 60 years of age, having at least a 12.5 hemoglobin level, and weighing above 50 kg to be qualified for donation. Persons with high-risk behavior or any other deferral cause were excluded from the study. The study evaluated the annual prevalence of HCV, HBV, and HIV in blood donors at the respective blood banks and overall.

The ethical committee of the SBTA approved the study proposal and the protocol under the 0002 approval number. Informed written consent, medical and socio-demographic histories, and a donor history questionnaire containing the previous history of blood transfusions and risky sexual behaviors of donors were collected from each enrolled participant.

ABO and Rhesus (Rh) blood typing were carried out by using monoclonal blood grouping antiserum, which included anti-A, anti-B, anti-AB, and anti-D. All donations were tested according to the standardized screening test algorithms for hepatitis B surface antigen (HBsAg), anti-HCV, and anti-HIV. Screening was performed by enzyme-linked immunosorbent assay (ELISA) or chemiluminescent immunoassay (CLIA). All reactive samples were repeated in duplicate. The positive blood units were discarded and the donors were informed confidentially about their screening results. Statistical analysis was carried out via Statistical Package for Social Sciences (SPSS) software version 22 (Armonk, NY: IBM Corp). The frequency was calculated annually for each of the TTI and then cumulative analysis was done. A p-value of <0.05 was considered to be significant. 

## Results

The results of serological screening tests for HBV, HCV, and HIV performed from January 2018 to December 2022 revealed a continuously increasing trend of all infections. Among 4,199,195 donors 3,821,268 (91%) were replacement donors while only 377,927 (9%) were voluntary donors with 3,870,598 (92.2%) male donors and only 328,597 (7.8%) were females. Whereas with regard to donors’ age, most of them, i.e. 2,664,648 (63%), fall in the 29-39 years age group (Table 1).

**Table 1:** Age and sex distribution of donors

Age group	Gender		Total
	Male	Female	
18-28	256,789	85,423	342,212 (8.1%)
29-39	2,578,303	86,345	2,664,648 (63%)
40-50	512,531	79,456	591,987 (14.6%)
51-60	522,975	77,373	600,348 (14.3%)
Total	3,870,598	328,597	4,199,195 (100.0%)

Among 4,199,195 donors screened, 159,665 (3.80%) were seropositive for HbsAg, anti-HCV, and anti-HIV. The overall seroprevalence of HbsAg, anti-HCV, and anti-HIV among blood donors of 182 blood banks, for five years, were 22,822 (2.78%), 33,794 (3.82%), 28,161 (3.65%), 36,958(4.15%), and 37,930 (4.04%), respectively.

**Figure 1:**
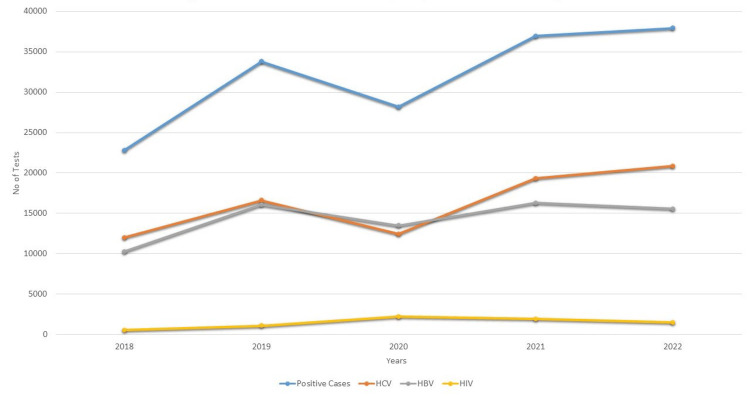
Seroprevalence and trends of HBV, HCV, and HIV over five years HBV: hepatitis  B virus; HCV: hepatitis C virus; HIV: human immunodeficiency virus.

The total number of blood donations in the blood bank showed a progressive increase from 22,822 donors in 2018 to 937,039 donors in 2022, with an increase of 14.21% blood donors in total. Overall, from 2018 to 2022, out of a total of 4,199,195 donors screened, 81,266 (1.94%) tested positive for HCV, 71,688 (1.7%) tested positive for HBV, and 6,711 (0.15%) tested positive for HIV. The total number of positive cases across all three infections was 159,665 (3.80%). The distribution of seroprevalence of HbsAg, anti-HCV, and anti-HIV antibodies according to the years is shown in Figure 1.

The prevalence of HCV was observed to range from 12,007 (1.46%) in 2018 to 20,863 (2.22%) in 2022. There was a 38.4% increase in frequency initially from 2018 to 2019, a slight decrease in cases from 2019 to 2020, most likely due to the COVID wave, and a surprising 55.6% increase in HCV cases from 2020 to 2021. A 7.9% increase was observed from 2021 to 2022 with 20,863 (2.22%) positive cases in 2022.

The prevalence of HBV among blood donors in 2018 was 10,270 accounting for 1.25% of the total screened. The positive cases increased gradually during 2018 and 2019 with a surprising 56.6% increase. However, it decreased from the year 2019 to 2020 with a 16% decrease. There was a percentage rise between 2020 and 2021 and then there was a 4.5% decrease in the following year 2022. The percentages represent the change in the number of positive cases of each infection compared to the previous year. Notably, hepatitis B vaccination is available in Pakistan.

The prevalence of HIV among blood donors in 2018 was 545, accounting for 0.07% of the total screened. The positive cases increased rapidly in 2019 and 2020. In 2021, there was a 40% decrease in cases from 2234 to 1329. There was a 13% rise between 2021 and 2022. The percentages represent the change in the number of positive cases of each infection compared to the previous year. For all major TTIs, there was an increasing trend over these years (Table 2).

**Table 2:** Seroprevalence of HBsAg, anti-HCV, and anti-HIV among blood donors over the years HCV: hepatitis C; HBV: hepatitis B; HBsAg: hepatitis B surface antigen; HIV: human immunodeficiency virus; SD: standard deviation. p-Values are original. Significant p-value ≤ 0.05. Mean, SD, and p-value: Not applicable in the total screened column.

Years	HCV positive (%)	HBV positive (%)	HIV positive (%)	Total positive (%)	Total screened
2018	12,007 (1.46)	10,270 (1.25)	545 (0.07)	22,822 (2.78)	820,493
2019	16,617 (1.9)	16,082 (1.8)	1095 (0.12)	33,794 (3.82)	870,662
2020	12,437 (1.57)	13,490 (1.82)	2234 (0.26)	28,161 (3.65)	705,550
2021	19,342 (2.20)	16,287 (1.80)	1329 (0.15)	36,958 (4.15)	865,451
2022	20,863 (2.22)	15,559 (1.66)	1508 (0.16)	37,930 (4.04)	937,039
Total	81 266 (1.94)	71 688 (1.7)	6711 (0.15)	159,665 (3.80)	4,199,195
Mean	16253.2	14337.6	1342.2	31,933	‘-‘
SD	4406.15	3929.19	1408.44	2345.02	‘-‘
p-Value	0.000	0.000	0.000	0.000	‘-‘
Chi-square	52600.62				

## Discussion

The seroprevalence of HBV, HCV, and HIV among healthy donor populations mostly from the Sindh area of Pakistan was evaluated in the study. The ideal population for the seroprevalence of the infectious studies is the general population; however, this is not feasible, so a healthy donor population was studied in this research.

Transfusion services in the country are not quality guaranteed or standardized because of poor administration and regulation. The blood transfusion authorities, which regulate inspection, licensing, and data management in all provinces, have been warned but are still in their initial stages. Pakistan's health level falls well short of the international norms that other countries strive toward. As a result, blood transfusions remain a significant risk factor for the development of HCV and other viral diseases [12]. Replacement donors constitute the largest portion of blood donors in Pakistan; in the current study, among 4,199,195 donors, 3,821,268 (91%) were replacement donors while only 377,927 (9%) were voluntary donors, which is comparable to other local studies in which replacement donors made up the bulk of blood donations with the goal of assisting a friend, relative, or acquaintance who required a blood transfusion [13]. The age range of 18 to 30 years produced the most donors; in the current study and past reports, a tendency of this nature was seen. Additionally, given the low number of female donors identified by the present study, efforts should be undertaken to boost the number of female donors [12-14]. Several developed nations have outlawed the use of paid blood donors to lessen the risk of diseases transferred through transfusions. Since paid blood donations were outlawed in China until 1998, many blood banks currently only accept volunteer contributions. With the use of this technique, the prevalence of HCV in China's general population has decreased from 8.68% in 1990 to 3.2% in 2010. India, Pakistan's neighbor, put into effect a law banning any paid contributions in 1998, which effectively decreased the incidence of TTIs within the country [15]. It has been reported that the percentage prevalence of HBV, HCV, and HIV in India among blood donors reduced from 1.62%, 1.85%, and 1.16% (in 2004) to 0.92%, 0.52%, and 0.21% (in 2009), respectively. The prevalence of these viral infections decreased significantly in our neighboring countries in recent years [14,16].

The study findings showed that the prevalence of HCV ranged from 1.46% in 2018 to 2.22% in 2022, with significant fluctuations over the years. The prevalence of HBV showed a similar pattern, ranging from 1.25% in 2018 to 1.66% in 2022. HIV prevalence exhibited a more consistent trend, with 0.07% in 2018 and 0.16% in 2022. These percentages represent the change in the number of positive cases compared to the previous year. HBV, HCV, and HIV were found to be prevalent in a local study in Karachi city with a 1.84%, 1.7%, and 0.04% reactivity ratio, respectively. The results of the current study are consistent with other local studies, where the total number of positive donors in 2020 is less as compared to the positive detected cases in 2019 [13]. According to Hassan Abbas et al., the overall seroprevalence of HBsAg, anti-HCV, and anti-HIV among blood donors in Islamabad was 2.35%, 3.26%, and 0.017%, respectively [17]. The current findings demonstrate that in 2018, the overall seroprevalence of HBsAg, anti-HCV, and anti-HIV among blood donors in Sindh blood banks was 1.46%, 1.25%, and 0.07%, respectively, while in 2022, the overall prevalence rose to 2.22%, 1.66%, and 0.16%, respectively. This upward trend was statistically significant in previous studies when consecutive years were compared between 2018 and 2022 [18,19].

The seroprevalence rate of HBV and HCV among blood donors in the southern part of Pakistan (Karachi) reported by Kakepoto et al. was almost the same (HBsAg 2.28% and anti-HCV 1.18%) as reported by us and by another study (HbsAg 5.0% anti-HIV 2.4%) reported from the same area [19]. However, there are obvious differences between data reported by studies conducted in Punjab and other provinces. Mujeeb et al. have however observed that the positivity for HCV was directly related to the level of literacy; thus, a higher number of educated donors visiting AKUH could be responsible for the decreased prevalence rate of HCV in their data [20]. The WHO has approximated that there are around 350 million individuals affected by chronic HBV infection and 170 million individuals affected by chronic HCV infection globally [21,22]. HBV is responsible for an estimated 563,000 deaths each year, while HCV results in approximately 366,000 deaths annually [23]. Among countries heavily impacted by these diseases, Pakistan stands out due to its large population of 240 million and its intermediate-to-high rates of infection, making it one of the most severely affected nations. In Pakistan, the prevalence in children is around 2.1%, and in adults, it varies between 0.5% and 31.9% [24].

In Pakistan, where there is only a nascent culture of charity contributions, a large dependence on replacement, and no systematic screening, infection risks are higher since relative substitute donors are more likely than voluntary donors to spread transfusion-transmissible illnesses.

The current situation is dire since blood donors in many places in Pakistan are not routinely screened for HBV and HCV. To further record the prevalence of seroreactivity of HBV, HCV, and HIV in the general population and to test the hypothesis that has been proposed in the present study, comprehensive epidemiological investigations are required. These will aid in the design of studies to clarify the natural history of HCV in our population, including mechanisms of transmission besides parenteral transmission. A larger population that has received the hepatitis B vaccine could further reduce the disease's prevalence. It is crucial to take necessary actions to maintain low HIV prevalence rates. This study also signifies the importance of proper testing; as CLIA was used in this study, the sensitivity and specificity are still far better than ICT or ELISA methods; however, only NAT testing can aid in diagnosing the actual percentage of these infections in donors and can make blood 100% safe for transfusion.

Limitations

The most significant limitation of our study is the use of a screening test instead of any confirmatory test in the study; moreover, as it was from all blood banks registered by the SBTA of Sindh, there were different types of analyzers used in the study. Though the method used was the same CLIA, different analyzers have different sensitivity and specificity as well as different cutoff values.

The other important limitation of the study is that the data analysis was conducted on the data provided by different blood banks to SBTA and no secondary verification could be done.

Thirdly, we have not analyzed the data region-wise/district-wise, so the actual frequency per area could not be identified in the study.

## Conclusions

The study highlights a concerning increase in the prevalence of HCV, HBV, and HIV among blood donors in Sindh, Pakistan, over five years. It underscores the importance of continued surveillance, prevention, and intervention strategies to address these infections. Further research is needed to identify the risk factors and develop targeted interventions for reducing the burden of these infections among blood donors and the general population. There should be centralized blood collection systems having better personnel and equipment, and non-remunerated voluntary blood donations must be strongly encouraged. All these, however, require strong governmental devotion, multisector loyalty, and a powerful surveillance system.
